# Evaluation of biventricular longitudinal myocardial function in normal fetuses at different gestational ages using ultrasonic velocity vector imaging

**DOI:** 10.3389/fped.2022.920966

**Published:** 2022-08-08

**Authors:** Min Hou, Liu Liu, Jun-Min Xie, Xiao-Jing Duan, Xiao-Lan Lv, Xiao-Qing Li, Qin Zhang

**Affiliations:** ^1^Department of Ultrasonography, Affiliated Hospital of Hebei University, Baoding, China; ^2^Department of Ultrasonography, Baoding Maternal and Child Health Hospital, Baoding, China; ^3^Department of Cardiology, Affiliated Hospital of Hebei University, Baoding, China; ^4^Department of Ultrasonography, Baoding No.1 Central Hospital, Baoding, China; ^5^Department of Ultrasonography, Gaoyang County Hospital, Baoding, China

**Keywords:** strain, strain rate, ultrasonic velocity vector imaging, fetus, biventricular longitudinal myocardial function

## Abstract

**Objective:**

This study aimed to evaluate biventricular myocardial function and biventricular longitudinal global myocardial function of fetuses at different gestational weeks using ultrasonic velocity vector imaging.

**Methods:**

A total of 127 pregnant women were enrolled and divided into five groups according to the gestational age of their fetuses. The velocity, strain, and strain rate of the left and right ventricles were measured, and these biventricular parameters were compared between the groups. The global parameters of the biventricular myocardium were also compared.

**Results:**

A pairwise comparison revealed that the differences in biventricular velocity and strain rate between groups in adjacent gestational weeks were not statistically significant (*P* > 0.05), but velocity increased with gestational age. A comparison of fetal longitudinal global myocardial parameters revealed that the global velocity, strain, and strain rate of the right ventricle were higher than those of the left ventricle, and the differences were statistically significant (*P* < 0.05) in all groups.

**Conclusion:**

The peak velocities of the fetal left and right ventricles increased with gestational age, but the global strain and strain rate did not, suggesting that fetal myocardial function is mature and constant in the middle and late stages of pregnancy and can more reliably reflect myocardial deformation. The peak systolic velocity, global strain, and peak strain rate of the right ventricle were higher than those of the left ventricle, suggesting that the right ventricle dominates longitudinal systolic movement from the second trimester of pregnancy.

## Introduction

Fetal heart examination, including the evaluation of cardiac structure and function, is a key part of pregnancy screening, and the development of ultrasound technology has allowed the diagnosis of fetal cardiac structural abnormalities to mature. M-mode ultrasound, two-dimensional ultrasound, color Doppler ultrasound, and spectral Doppler ultrasound, among other methods, are traditionally used to analyze and evaluate the global function of the fetal heart according to various parameters, such as ejection and shortening fractions, which are calculated according to the end systolic and end diastolic volume of the left ventricle (1–3). However, because of the influence of various factors, such as the differing technical experience of operators, patient age, bradycardia or tachycardia, and left ventricular load change, the repeatability of the examination results is poor.

Velocity vector imaging (VVI), an echocardiographic technique based on two-dimensional gray scale, was first applied to fetal echocardiography in 2007 (4). VVI has a low dependence on angle and a good signal-to-noise ratio (5, 6). It can also automatically determine the center of motion with high repeatability (7) and can quantitatively provide the peak velocity, strain, and strain rate of the ventricular wall in the long axis, short axis, and axial direction of the segmental myocardium (8). Tissue velocity is expressed in the form of a vector, which displays the movement direction, velocity magnitude, movement distance, velocity gradient change value, and other information related to tissue structure, with the velocity magnitude of tissue movement represented by the length of the vector (the length of the arrow) and the velocity direction of tissue movement represented by the direction of the vector (the direction indicated by the arrow). VVI is less affected by the angle of sound beams and can overcome the impacts of respiration, global cardiac motion, cardiac rotation, and adjacent myocardial segment mortality or restriction.

Common parameters used to describe myocardial function include strain and strain rate. Strain refers to the deformation ability of local myocardial tissue after stress. The strain rate is the rate of deformation, referring to the degree of deformation in unit time. It is less affected by the displacement caused by heart rotation and swing and the traction effect of adjacent tissues and can better reflect the function of the local myocardium (9).

This study aimed to evaluate the biventricular myocardial function and biventricular longitudinal global myocardial function of fetuses at different gestational weeks using ultrasonic VVI.

## Materials and methods

### Study participants

A total of 127 pregnant women undergoing examination between January 2016 and April 2018 were enrolled in this study, and their fetuses were divided into five groups according to gestational age. Written informed consent was obtained from all the participants' parents.

Inclusion criteria: (1) the patient had no family history of congenital heart disease; (2) the patient had no infectious diseases; (3) the patient had no systemic diseases, such as hypertension or diabetes; (4) the patient was receiving no medication; (5) the patient had no radiation history; (6) the patient had an accurate menstrual history or the gestational age of the fetus was consistent with the gestational week confirmed by an early pregnancy ultrasound. All the enrolled fetuses were a singleton pregnancy with no cardiac structural malformation or arrhythmia.

### Research instruments

The Voluson E8/E10 color Doppler ultrasound diagnostic apparatus (GE Healthcare, China) was used in this study, with a probe frequency of 2–5 MHz. Two-dimensional strain VVI analysis software (TomTec, Germany) was used offline for the post-processing analysis.

### Research methods and detection indicators

**Figure 1:**
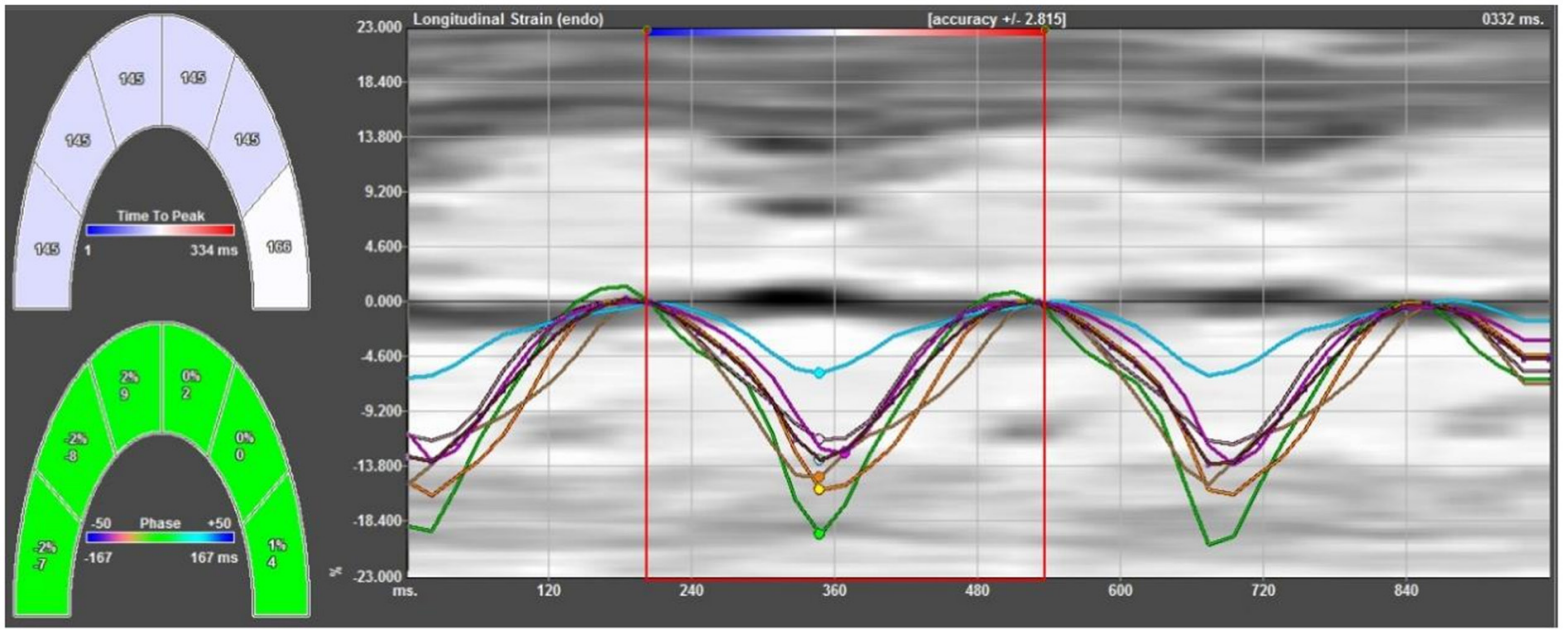
Strain curves of all segments of the left ventricular lateral wall and left ventricular side of the ventricular septum. The strain curve has a negative single peak in the whole cardiac cycle; in the systolic phase, the myocardium shortens longitudinally, developing from zero to negative, and reaches its maximum strain at the end of the systolic phase, gradually returning to zero in the diastolic phase.

The data of the fetal apical four-chamber view were stored in the computer to ensure that the endocardial image was clear and legible. The fetal left and right ventricular walls were divided into 12 segments, namely the basal segment, middle segment, and the apical segment of the left and right ventricles and their corresponding ventricular septal side. The ultrasound apparatus was then moved offline, and the image was imported into the VVI analysis software. The static single-frame image in which the endocardial interface was most clearly displayed was selected. The ventricular boundary was then manually traced along the endocardial interface, and the start and end points were located at the root of the tricuspid valve and mitral valve ring, respectively. Subsequently, the velocity, strain, and strain rates of the whole and segmental myocardium were automatically obtained (Figure 1). The average value was calculated from three cardiac cycles.

### Statistical methods

Data were analyzed using SPSS 22.0 statistical software. Measurement data first underwent a test of normality. Normally distributed measurement data were expressed as mean ± standard deviation ($\bar{x}\;\pm$ SD). A comparison between fetuses in the same gestational age group was conducted using a *t*-test. A comparison of parameter values between segments was conducted using a paired *t*-test, and a comparison of parameter values between the left and right ventricles was conducted using an independent *t*-test. The correlation between parameters and gestational age was analyzed, and the linear equation was fitted. A reliability analysis of inter-observer and intra-observer measurements was conducted. *P* < 0.05 was considered statistically significant.

## Results

An offline analysis involving 127 fetal examination images was conducted, and 120 cases were successfully analyzed, with a success rate of 94.5%. All the fetuses were divided into five groups according to gestational age: 16–19^+6^ weeks (group 1, *n* = 14), 20–23^+6^ weeks (group 2, *n* = 36), 24–27^+6^ weeks (group 3, *n* = 37), 28–31^+6^ weeks (group 4, *n* = 20), and 32–35^+6^ weeks (group 5, *n* = 13).

### Comparison of parameters of the fetal ventricular longitudinal global myocardium between different gestational ages

#### Velocity

**Figure 2:**
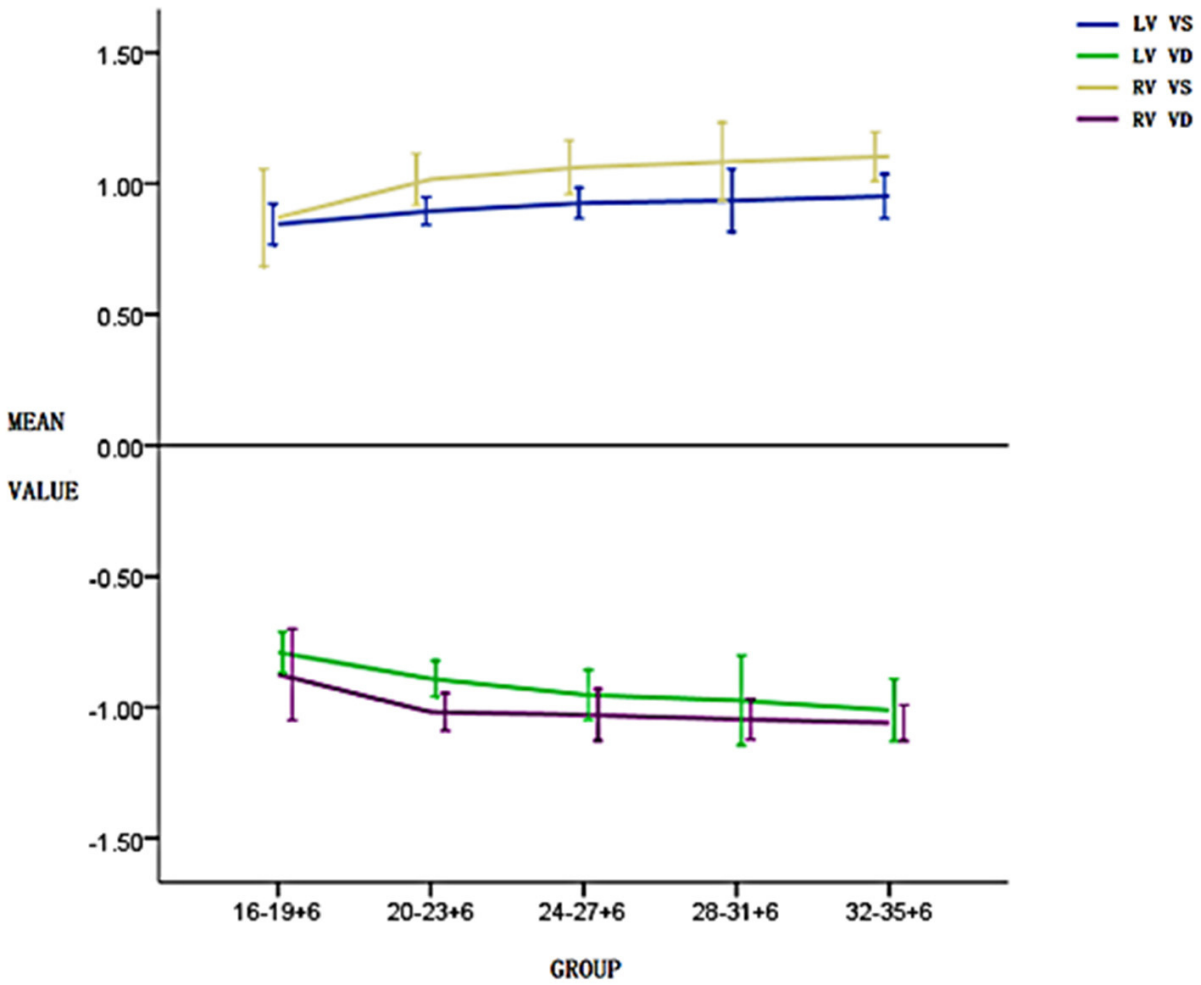
Changes in the left and right ventricular systolic and diastolic global peak velocities in five pregnancy groups. The global peak velocity gradually increases with the increase in gestational weeks and the global peak velocity of the right ventricle in both systolic and diastolic phases is higher than that of the left ventricle.

A pairwise comparison between adjacent gestational age groups revealed no significant differences in systolic and diastolic longitudinal global peak velocities (*P* > 0.05). However, the general trend in global peak velocity was a gradual increase with gestational age (Figure 2), and the global peak VVI parameter values of the right ventricle were larger than those of the left ventricle in both systolic and diastolic phases (Figure 2, Tables 1, 2).

**Table 1:** Global peak velocity, strain and strain rate of fetal left ventricular myocardium in 5 gestational weeks groups in normal pregnancy.

**Group**	**Vs**	**Vd**	**S**	**SRs**	**SRd**
**(weeks)**	
16–19^+6^	0.85 ± 0.13	−0.79 ± 0.13	−16.62 ± 6.40	−1.68 ± 0.77	1.70 ± 0.65
20–23^+6^	0.90 ± 0.16	−0.89 ± 0.20	−17.50 ± 3.72	−1.66 ± 0.67	1.58 ± 0.42
24–27^+6^	0.93 ± 0.18	−0.95 ± 0.29	−15.37 ± 7.44	−1.45 ± 1.00	1.62 ± 0.72
28–31^+6^	0.94 ± 0.26	−0.97 ± 0.37	−15.38 ± 6.16	−1.34 ± 0.68	1.39 ± 0.64
32–35^+6^	0.95 ± 0.14	−1.01 ± 0.20	−14.34 ± 6.81	−1.39 ± 0.82	1.44 ± 0.57

*Vs, peak systolic velocity (cm/s)*.

*Vd, peak diastolic velocity (cm/s)*.

*S, strain (%)*.

*SRs, systolic strain rate (S^−1^)*.

*SRd, diastolic strain rate (S^−1^)*.

**Table 2:** Global peak velocity, strain and strain rate of fetal right ventricular myocardium in 5 gestational weeks groups in normal pregnancy.

**Group**	**Vs**	**Vd**	**S**	**SRs**	**SRd**
**(weeks)**	
16–19^+6^	0.87 ± 0.32	−0.87 ± 0.30	−19.90 ± 10.21	−1.89 ± 0.98	2.06 ± 0.94
20–23^+6^	1.02 ± 0.29	−1.02 ± 0.21	−20.33 ± 10.52	−2.27 ± 1.23	2.16 ± 0.88
24–27^+6^	1.06 ± 0.31	−1.03 ± 0.30	−19.52 ± 11.12	−1.98 ± 1.00	2.15 ± 0.87
28–31^+6^	1.08 ± 0.32	−1.05 ± 0.17	−19.84 ± 9.73	−1.72 ± 0.90	1.73 ± 0.80
32–35^+6^	1.10 ± 0.15	−1.06 ± 0.11	−16.36 ± 11.37	−2.28 ± 1.13	1.83 ± 1.19

*Vs, peak systolic velocity (cm/s)*.

*Vd, peak diastolic velocity (cm/s)*.

*S, strain (%)*.

*SRs, systolic strain rate (S^−1^)*.

*SRd, diastolic strain rate (S^−1^)*.

#### Strain

A pairwise comparison between adjacent gestational age groups revealed no significant differences in the longitudinal peak strain of the fetal left ventricle or right ventricle (*P* > 0.05; Tables 1, 2).

#### Strain rate

A pairwise comparison between adjacent gestational age groups revealed no significant differences in the longitudinal global peak strain rate of the fetal left ventricle and right ventricle in both systolic and diastolic phases (*P* > 0.05; Tables 1, 2).

### Comparison of longitudinal global myocardial velocity vector imaging parameters between fetal left and right ventricles

#### Velocity

In all groups, in both systolic and diastolic phases, the global peak velocity of the right ventricle was significantly higher than that of the left ventricle (*P* < 0.05; see Table 3).

**Table 3:** Six segments and global velocity, strain and strain rate of left and right ventricular myocardium of normal fetal heart in both systolic and diastolic phases.

	**Basal segment**		**Middle segment**		**Cardiac apical segment**		
	**Side wall**	**Ventricular**	**Side wall**	**Ventricular**	**Side wall**	**Ventricular**	**Global**
	**(free wall)**	**septal side**	**(free wall)**	**septal side**	**(free wall)**	**septal side**	**value**
**Left ventricle**							
Vs	1.43 ± 0.54^*^	1.32 ± 0.68^*^	0.95 ± 0.57^*^	0.90 ± 0.51^*^	0.55 ± 0.26	0.44 ± 0.30	
Vd	−1.57 ± 0.49^*^	−1.29 ± 0.71^*^	−0.96 ± 0.49^*^	−0.88 ± 0.61^*^	−0.57 ± 0.27	−0.45 ± 0.33	
S	−18.00 ± 12.55	−14.77 ± 9.79	−17.34 ± 9.37	−13.59 ± 9.79	−17.95 ± 12.50	−14.73 ± 9.99	
SRs	−1.84 ± 1.25	−1.54 ± 0.90	−1.63 ± 1.28	−1.49 ± 0.89	−1.85 ± 1.25	−1.59 ± 1.06	
SRd	1.75 ± 1.06	1.59 ± 0.81	1.58 ± 0.95	1.48 ± 1.01	1.68 ± 0.85	1.56 ± 0.88	
**Right ventricle**							
Vs	1.57 ± 0.66^*^	1.45 ± 0.64^*^	1.10 ± 0.48^*^	0.97 ± 0.60^*^	0.53 ± 0.30	0.43 ± 0.21	
Vd	−1.73 ± 0.79^*^	−1.67 ± 0.71^*^	−1.19 ± 0.65^*^	−0.98 ± 0.52^*^	−0.57 ± 0.45	−0.49 ± 0.27	
S	−19.59 ± 13.29	−19.42 ± 12.79	−17.07 ± 11.99	−18.73 ± 13.81	−18.16 ± 11.02	−17.39 ± 11.07	
SRs	−1.87 ± 1.19	−2.00 ± 1.73	−1.66 ± 1.36	−1.83 ± 1.46	−1.76 ± 1.15	−1.78 ± 1.21	
SRd	2.08 ± 1.19	2.13 ± 1.73	2.03 ± 1.20	1.97 ± 1.46	1.91 ± 1.13	1.87 ± 1.13	

* *Represents left ventricle and right ventricle*.

*Vs, peak systolic velocity (cm/s)*.

*Vd, peak diastolic velocity (cm/s)*.

*S, strain (%)*.

*SRs, systolic strain rate (S^−1^)*.

*SRd, diastolic strain rate (S^−1^)*.

#### Strain

The peak systolic strain of the right ventricle was significantly stronger than that of the left ventricle (*P* < 0.05; see Table 3).

#### Strain rate

In both systolic and diastolic phases, the global peak strain rate of the right ventricle was significantly higher than that of the left ventricle (*P* < 0.05; see Table 3).

### Velocity vector imaging parameter repeatability test

Twenty participants were randomly selected for a repeatability test.

#### Intra-observer consistency test

The VVI parameters in the participants' images were measured repeatedly by the same echocardiographist at an interval of more than 15 days. The intraclass correlation coefficients (ICCs) for segmental peak velocity, strain, and strain rate were 0.89, 0.87, and 0.83, respectively, and the ICCs for global peak velocity, strain, and strain rate were 0.88, 0.86, and 0.89, respectively.

#### Inter-observer consistency test

The above parameters were measured again by another doctor with equivalent experience. The ICCs for segmental peak velocity, strain, and strain rate were 0.89, 0.90, and 0.87, respectively, and the ICCs for global peak velocity, strain, and strain rate were 0.85, 0.86, and 0.88, respectively.

## Discussion

The accurate assessment of fetal cardiac function is helpful for confirming and improving our understanding of the development of fetal diseases and their impact on the fetal heart. Echocardiographic myocardial strain measurements have been demonstrated to be useful in assessing mild myocardial dysfunction and segmental ventricular wall motion abnormalities (10–14).

In VVI, velocity (displacement) is represented as a vector; in addition to velocity, it also indicates direction. Therefore, different spatial components can be verified along the *x, y*, and *z* axes, and the longitudinal, circumferential, and axial spatial components can be measured along the anatomical coordinates of the cardiac cavity; all of these parameters are closely related to the mechanical properties of the myocardium. The key advantage of using strain and strain rate relative to displacement is that they are independent of translational motion and reflect segmental function. Generally, strain describes the amplitude and direction of local myocardial shortening or prolongation; global strain, or more accurately, global longitudinal strain or global circumferential strain, usually refers to the average longitudinal or circumferential strain of the whole myocardium, which can approximate the average strain of the myocardium of each ventricular wall segment.

With the growth and development of the fetus, the heart volume increases, leading to an increase in longitudinal myocardial displacement. When the cardiac cycle becomes relatively constant [the development of cardiac function tends to mature in the second trimester of pregnancy (15)], the instantaneous displacement of the cardiac ventricle increases in both systolic and diastolic phases, resulting in the acceleration of peak velocity with the increase in gestational age. The findings of the present study are therefore consistent with those of previous studies.

In the present study, the changes identified in global strain, strain rate, and gestational age are consistent with the findings of previous studies (16, 17) in that they did not change with the increase in gestational age. Strain is not related to time; thus, it can more reliably reflect the deformation of the myocardium, suggesting that fetal cardiac function is mature and constant in the middle and late stages of pregnancy. Previous studies have also revealed that the global longitudinal strain of the fetal left and right ventricles decreases in the last 4 weeks of pregnancy (18), with only the right ventricular strain changing (19). These differences may be caused by the application of different ultrasound systems, myocardial deformation analysis software packages, and speckle tracking methods to calculate parameters.

The strain and strain rate of the left and right ventricles in the present study did not change with the increase in gestational age, and the comparison between the five gestational age groups revealed no statistically significant differences in these parameters. According to the following strain rate formula,

$$\begin{array}{l} SR=\frac{S}{\Delta t}=\frac{\frac{\Delta L}{L_{0}}}{\Delta t}=\frac{\frac{\Delta L}{\Delta t}}{L_{0}}=\frac{\Delta v}{L_{0}}=\frac{v_{1}-v_{2}}{L_{0}} \end{array}$$

(*S*, strain; *SR*, strain rate; Δ*t*, time change; Δ*v*, velocity change; *L*_0_, initial length), strain is stable and unchanged in the middle and late stages of pregnancy; if the fetal heart rate fluctuates within the normal range, then the strain rate does not change with the increase in gestational age.

The present study also revealed that in all the groups, the peak systolic global velocity of the right ventricle was higher than that of the left ventricle, and the longitudinal global strain and peak strain rates of the right ventricle were higher than those of the left ventricle. The study also demonstrated that, during the fetal period, the contraction of the right ventricle mainly occurs in the longitudinal direction. The direction of myocardial fibers determines the pump function of the myocardium (20, 21). Myocardial fibers can be divided into three layers: the inner longitudinal bunch, medium ring, and outer oblique layer. Although the inner and outer layers of the left and right ventricles have longitudinal and/or oblique fibers, the middle layer's annular fibers only exist in the left ventricle. Therefore, the right ventricle is dominated by longitudinal and/or oblique fibers, whereas the left ventricle has both longitudinal and/or oblique fibers and annular fibers. Compared with the left ventricle, the fiber distribution makes the longitudinal shortening of the right ventricle more obvious during cardiac contraction. Therefore, from the second trimester of pregnancy, the right ventricle dominates longitudinal systolic movement.

This study has some limitations. An evaluation of the ventricular circumferential myocardial function was not performed because the success rate of obtaining the appropriate short-axis image is very low, and the intra-observer and inter-observer consistency tests were poor. Moreover, this process is time-consuming and therefore the clinical application of general fetal evaluation may be limited.

## Conclusion

This study revealed that, in normal fetuses, the longitudinal global myocardial peak systolic velocity and peak diastolic velocity of the left and right ventricles increased with gestational age, whereas strain, systolic strain rate, and diastolic strain rate did not change with the change in gestational age, suggesting that they remain stable in the middle and late stages of pregnancy. Furthermore, the global myocardial VVI parameters of the fetal right ventricle were higher than those of the left ventricle, confirming that, from the second trimester of pregnancy, the right ventricle dominates longitudinal systolic movement.

## Data availability statement

The original contributions presented in the study are included in the article/supplementary material, further inquiries can be directed to the corresponding author.

## Ethics statement

The studies involving human participants were reviewed and approved by the Ethics Committee of Affiliated Hospital of Hebei University. The patients/participants provided their written informed consent to participate in this study.

## Author contributions

MH, LL, and J-MX: conception and design of the research. MH: acquisition of data. MH, LL, J-MX, X-JD, X-LL, X-QL, and QZ: analysis and interpretation of the data. MH, J-MX, X-LL, and X-QL: statistical analysis. MH, LL, J-MX, and X-JD: writing of the manuscript. J-MX: critical revision of the manuscript for intellectual content. All authors read and approved the final draft.

## Conflict of interest

The authors declare that the research was conducted in the absence of any commercial or financial relationships that could be construed as a potential conflict of interest.

## Publisher's note

All claims expressed in this article are solely those of the authors and do not necessarily represent those of their affiliated organizations, or those of the publisher, the editors and the reviewers. Any product that may be evaluated in this article, or claim that may be made by its manufacturer, is not guaranteed or endorsed by the publisher.

## Abbreviations

VVI: Velocity vector imaging
Vs: Peak systolic velocity
Vd: Peak diastolic velocity
S: Strain
SRs: Systolic strain rate
SRd: Diastolic strain rate
ICC: Intraclass correlation coefficient.

## References

[1] Herling L Johnson J Ferm-Widlund K Bergholm F Elmstedt N Lindgren P. Automated analysis of fetal cardiac function using color tissue Doppler imaging in second half of normal pregnancy. Ultrasound Obstetr Gynecol. (2018) 2018:53. 10.1002/uog.19037 29484743

[2] Germanakis I Gardiner H. Assessment of fetal myocardial deformation using speckle tracking techniques. Fetal Diagn Ther. (2012) 32:39–46. 10.1159/000330378 22626849

[3] Butz T van Buuren F Mellwig KP Langer C Oldenburg O Treusch KA. Echocardiographic tissue Doppler imaging analysis of the systolic and early diastolic velocities of the mitral annulus motion in hypertrophic cardiomyopathy and in top-level athletes. Ultraschall in der Medizin. (2012) 33:455–62. 10.1055/s-0029-1246069 21294072

[4] Younoszai AK Saudek DE Emery SP Thomas JD. Evaluation of myocardial mechanicsin the fetus by velocity vector imaging. J Am Soc Echocardiogr. (2008) 21:470–4. 10.1016/j.echo.2007.08.003 17904801

[5] Vieira MJ Teixeira R Gonçalves L Gersh BJ. Left atrial mechanics: echocardiographic assessment and clinical implications. J Am Soc Echocardiogr. (2014) 27:463–78. 10.1016/j.echo.2014.01.021 24656882

[6] Guo JP Wang Y Zhi G Zhang X Lin K. Predict value of time to peak of systolic velocity derived from velocity vector imaging on cardiac resynchronization therapy response in refractory heart failure patients. Chinese J Cardiovasc Dis. (2015) 43:806–10. 26652823

[7] Tang S Yuming M. The value of ultrasound in cardiac resynchronization therapy. Medical Recapitulate. (2015) 21:296–8. 10.3969/j.issn.1006-2084.2015.02.039

[8] Suursalmi P Ojala T Poutanen T Eerola A Korhonen P Kopeli T. Velocity vector imaging shows normal cardiac systolic function in survivors of severe bronchopulmonary dysplasia at six to 16 years of age. Acta Paediatr. (2017) 106:1136–41. 10.1111/apa.13860 28370347

[9] Sutherland GR Disalvo G Claus P D'hooge J Bijnens B. Strain and strain rate imaging:a new clinical approach to quantifying regiona myocardial function. Expert Rev Cardiovasc Ther. (2015) 13:853–66. 10.1586/14779072.2015.1056163 26058981

[10] Blanc J Stos B de Montalembert M Bonnet D Boudjemline Y. Right ventricular systolic strain is altered in children with sickle cell disease. J Am SocEchocardiogr. (2012) 25:511–7. 10.1016/j.echo.2012.01.011 22341367

[11] Di Salvo G Rea A Mormile A Limongelli G D'Andrea A Pergola V. Usefulness of bidimensional strain imaging for predicting outcome in asymptomatic patients aged ≤ 16 years with isolated moderate to severe aortic regurgitation. Am J Cardiol. (2012) 110:1051–5. 10.1016/j.amjcard.2012.05.039 22728004

[12] McCandless RT Minich LL Wilkinson SE McFadden ML Tani LY Menon SC. Myocardial strain and strain rate in Kawasaki disease. Euro Heart J Cardio Imaging. (2013) 14:1061–8. 10.1093/ehjci/jet041 23515218

[13] Petko C Hansen JH Scheewe J Rickers C Kramer HH. Comparison of longitudinal myocardial deformation and dyssynchrony in children with left and right ventricular morphology after the fontan operation using two dimensional speckle tracking. Congenit Heart Dis. (2012) 7:16–23. 10.1111/j.1747-0803.2011.00607.x 22176662

[14] Poterucha JT Kutty S Lindquist RK Li L Eidem BW. Changes in left ventricular longitudinal strain with anthracycline chemotherapy in adolescents precede subsequent decreased left ventricular ejection fraction. J Am Soc Echocardiogr. (2012) 25:733–40. 10.1016/j.echo.2012.04.007 22578518

[15] Wang XY Lian YJ Wang XF Tian M. Study of regional left ventricular longitudinal function in fetuses with gestational diabetes mellitus by velocity vector imaging. Echocardiography. (2016) 33:1228–33. 10.1111/echo.13238 27460645

[16] Liu MX Yu J Fu XX Wan W. Quantitative assessment of cardiac function in fetuses of women with maternal gestational thyroid dysfunction using VVI echocardiography. Med Sci Monit. (2015) 21:2956–68. 10.12659/MSM.894381 26427319 PMC4596453

[17] Kim SH Miyakoshi K Kadohira I Tanaka M Minegishi K Matsumoto T. Comparison of the right and left ventricular performance during the fetal development using velocity vector imaging. Early Hum Dev. (2013) 89:675–81. 10.1016/j.earlhumdev.2013.04.015 23707047

[18] Alsolai AA Bligh LN Greer RM Gooi A Kumar S. Myocardial strain assessment using velocity vector imaging in normally grown fetuses at term. Ultrasound Obstetr Gynecol. (2018) 52:352–8. 10.1002/uog.17549 28608400

[19] Chelliah A Dham N Frank LH Donofrio M Krishnan A. Myocardial strain can be measured from first trimester fetal echocardiography using velocity vector imaging. Prenat Diagn. (2016) 36:483–8. 10.1002/pd.4813 26991266

[20] Buckberg G Hoffman JIE. Right ventricular architecture responsible for mechanical performance: unifying role of ventricular septum. J Thorac Cardiovasc Surg. (2014) 148:3164–6. 10.1016/j.jtcvs.2014.05.044 24973008

[21] van Oostrum NHM de Vet CM Clur SB van der Woude DAA van den Heuvel ER Oei SG. Fetal myocardial deformation measured with two-dimensional speckle-tracking echocardiography: longitudinal prospective cohort study of 124 healthy fetuses. Ultrasound Obstetr Gynecol. (2022) 59:651–9. 10.1002/uog.24781 34558747 PMC9321172

